# Comparison of Fixed- and Variable-Loop Button Fixation in Arthroscopic Anterior Cruciate Ligament Reconstruction

**DOI:** 10.7759/cureus.24218

**Published:** 2022-04-17

**Authors:** Vijay Chandru, Santhosh M.S., Sujana Theja J.S., Rohit R Nair

**Affiliations:** 1 Orthopaedics and Traumatology, Jagadguru Sri Shivarathreeshwara Hospital, Jagadguru Sri Shivarathreeshwara Academy of Higher Education and Research, Mysuru, IND

**Keywords:** arthroscopic acl reconstruction, fixed loop devices, variable loop devices, graft laxity, lysholm knee score

## Abstract

Introduction

With the advent of fixed- and variable-loop suspensory fixation devices for arthroscopic anterior cruciate ligament (ACL) reconstruction, a maximum number of grafts can be placed within the femoral tunnel. Although several biomechanical studies have been conducted comparing these two devices, only a few comparative clinical studies are available. This study was conducted to compare the functional outcomes of arthroscopic ACL reconstruction using fixed-loop devices with those of variable-loop devices by determining their effect on graft laxity clinical assessment and patient-reported outcome scores.

Methodology

Out of 32 patients (27 males and five females) who underwent primary ACL reconstruction using tripled hamstring autograft, fixed- and variable-loop devices were used for 13 and 19 patients, respectively. Thirteen patients in each group were evaluated over a period of one year using the Lysholm knee score. Six patients in the variable-loop group had only six months of follow-up. Anterior drawer and Lachman tests were performed at six-month and one-year follow-ups, respectively.

Results

The mean ages of patients in the fixed- and variable-loop groups were 34.5$\pm$11 and 34.1$\pm$9.1 years, respectively. The Lysholm knee score at six weeks was fair in 7.7% of the patients in the fixed-loop group when compared to 52.6% of those in the variable-loop group (*p*<0.05). All the other parameters were comparable between the two groups. One patient in each group had ligament laxity at six-month and one-year follow-up, respectively.

Conclusion

This study showed no statistically significant difference in graft laxity or functional outcomes of arthroscopic ACL reconstruction with fixed- and variable-loop devices, except for a better patient-reported outcome score in the variable-loop group at six weeks of follow-up. Hence, there is a need for more comparative studies in this direction.

## Introduction

This article was previously presented as a poster at the 64th Annual Conference of Indian Orthopaedic Association (IOACON 2019) on 22 November 2019 and as a scientific paper at the 44th State Conference of Karnataka Orthopaedic Association (KOACON 2020) on 1 February 2020.

An anterior cruciate ligament (ACL) injury is the commonest knee injury seen by orthopaedic surgeons all over the world. Of all the factors contributing to a successful ACL reconstruction, graft fixation and tunnel incorporation are the most important determinants of post-operative knee stability [1].

A short femoral tunnel effectively reduces the length of the graft in it, thereby compromising on strength of the graft-bone tunnel construct [2]. Reduced graft length adversely affects healing following ACL reconstruction [3]. This has led to the emergence of suspensory fixation methods that accommodate a large number of grafts within the femoral tunnel. Suspensory fixation devices include fixed (closed)-loop systems and variable (adjustable)-loop systems.

In fixed-loop systems, the length of the graft tunnel and the size of the loop have a significant impact on the outcome of ACL reconstruction. When the graft tunnel is short, closed-loop systems may not function effectively. A long loop may result in insufficient graft within the femoral tunnel.

In variable-loop systems, pulling a series of sutures reduces the length of the loop and advances the graft to the femoral tunnel exit. This results in a larger contact area for bone-tendon healing, which in turn leads to better graft uptake. Hence, short tunnels do not preclude their usage. However, in biomechanical studies, these devices have exhibited more displacement than fixed-loop devices under lower loads than those occurring during early ACL rehabilitation [4]. The relevance of this finding in the clinical setup can be ascertained only if commensurate clinical studies are conducted comparing the two fixation methods.

The primary objective of this study was to compare the functional outcomes of arthroscopic ACL reconstruction using fixed-loop devices with that of variable-loop devices by determining their effect on clinically assessed graft laxity and functional outcome scores (the Lysholm knee score). The secondary objective was to evaluate the demographic distribution, mode, and MRI grading of ACL injuries and assess the pattern of associated injuries.

## Materials and methods

This was a prospective study involving 32 patients with an ACL tear who visited the outpatient department and later underwent arthroscopic ACL reconstruction in the Orthopaedics Department of JSS Medical College, JSS Academy of Higher Education and Research, Mysuru, between September 2017 and March 2019.

The inclusion criteria were as follows: (1) age: 18 years or older; (2) no other illnesses preventing them from ambulating normally; (3) clinical and MRI proved ACL insufficiency of grades 3 and 4 [5]; (4) no history of previous surgery or cruciate ligament damage in the affected knee; (5) patients with associated medial collateral ligament (MCL) or lateral collateral ligament (LCL) tear grade 1 or 2 with or without meniscal injury.

The exclusion criteria were as follows: (1) ACL rupture associated with tibial or femoral condyle fractures and tibial spine avulsion fractures; (2) ACL reconstruction with other modes of fixation on the femoral side; (3) posterior cruciate ligament injury; (4) evidence of osteoarthritis on plain radiograph.

Written informed consent for inclusion in the study was taken from all the patients. The treatment protocol was explained to them and they were briefed regarding their rights during the study. The treatment process had no adverse effects on the health of the participants, and the study was approved by the Institutional Ethical Committee of JSS Medical College, Mysuru (JSS Medical College Ethical Committee, date: 31/10/2017, no: JSSMC/PG/4700/2017-18).

All the patients were subjected to detailed history taking and clinical examination. Lachman and anterior drawer tests were performed on all the patients before getting an MRI done. If MRI revealed significant femoral or tibial condyle contusion, surgery was delayed by three weeks. Functional limitations of all the patients were assessed preoperatively using the Lysholm knee scoring scale. Post-anaesthesia pivot shift test was done for further confirmation of ACL tear. All the surgeries were performed by an experienced team of orthopaedic surgeons trained in arthroscopy.

Diagnostic arthroscopy was first done. Hamstring graft was then harvested, tripled, and pre-tensioned. The final graft size was 8 mm in all cases. The graft was then inserted through drill holes, assisted arthroscopically, and fixation was done with a fixed- or variable-loop device. The final femoral bone-tendon overlap length was 20 mm in all cases. The decision regarding the device to be used was made on-table based on the femoral tunnel length. If the tunnel length was less than 40 mm, variable-loop button fixation was done as a graft length of 20 mm had to be ensured within the tunnel (a 15-mm fixed-loop device is not available in our institute).

After the surgery, all the patients underwent physiotherapy according to the institutional ACL rehabilitation protocol. Patients were encouraged to bear weight as tolerated with crutches and range of motion (ROM) knee brace locked in extension, second postoperative day onwards. In the first two weeks, emphasis was placed on patella mobilization, flexion up to 90^o^ and full passive extension. Thereafter, progression to full knee flexion was encouraged by the end of six weeks. The active terminal extension was permitted only after six weeks. As and when patients demonstrated good quadriceps control, they were weaned off the brace and crutches. Patients were followed up at two weeks, four weeks, six weeks, three months, six months, nine months and 12 months. The objective and subjective evaluations were done with the Lysholm knee scoring scale. Graft laxity was assessed by a single individual, using Lachman and anterior drawer tests. These tests were performed on the affected as well as contralateral normal knees of patients, at six months and one year.

Data analysis was done using IBM SPSS Statistics for Windows, Version 21.0 (IBM Corp., Armonk, USA). Chi-square analysis was used for comparing the sex distribution, mode of injury, MRI grading of an ACL tear, associated meniscal injuries, Lachman and anterior drawer tests for graft laxity at six months and one year and the Lysholm knee score grades of the two groups (the Lysholm knee scores were graded as: excellent - 95-100; good - 84-94; fair - 65-83; poor - <64). An independent *t*-test was used to compare the age distribution and knee ROM between the groups. The number of days after the injury at which the patients in both groups underwent surgery was compared using the Mann-Whitney *U*-test. A *p*-value of <0.05 was considered statistically significant for all tests.

## Results

Of 32 patients (27 males and five females) who underwent arthroscopic ACL reconstruction, the fixed-loop device was used in 13 patients and the variable-loop device in 19 patients. Six patients in the variable-loop group were lost to follow-up after six months. All the others were followed up over a period of one year. Baseline patient characteristics are mentioned in Table 1. Both the groups were homogeneous for age, gender, mode of injury, median duration after injury at which surgery was performed and MRI grade of ACL injury.

**Table 1:** Baseline patient characteristics RTA, road traffic accident; MRI, magnetic resonance imaging.

Parameter		Fixed-loop group	Variable-loop group	*p*-value
Mean age		34.5$\pm$11 years	34.1$\pm$9.1 years	0.911
Gender	Male	12	15	0.3
	Female	1	4	
Mode of injury	Fall	6	6	0.54
	RTA	7	12	
	Assault	0	1	
Median duration after injury (in days)		90 (30-180)	60 (30-120)	0.62
MRI grade	Grade 3	4	5	0.7
	Grade 4	9	14	

**Figure 1:**
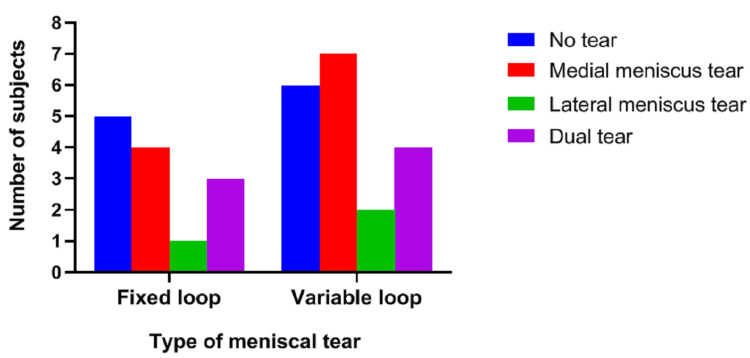
Associated medial and lateral meniscus tears

The distribution of patients with associated medial and lateral meniscus tears in each group is shown in Figure 1.

**Figure 2:**
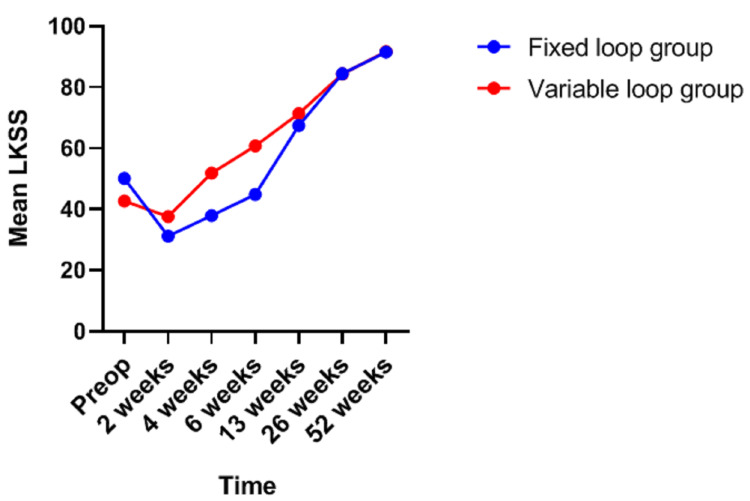
Mean Lysholm knee scoring scale Preop, preoperative; LKSS, Lysholm knee scoring scale.

**Figure 3:**
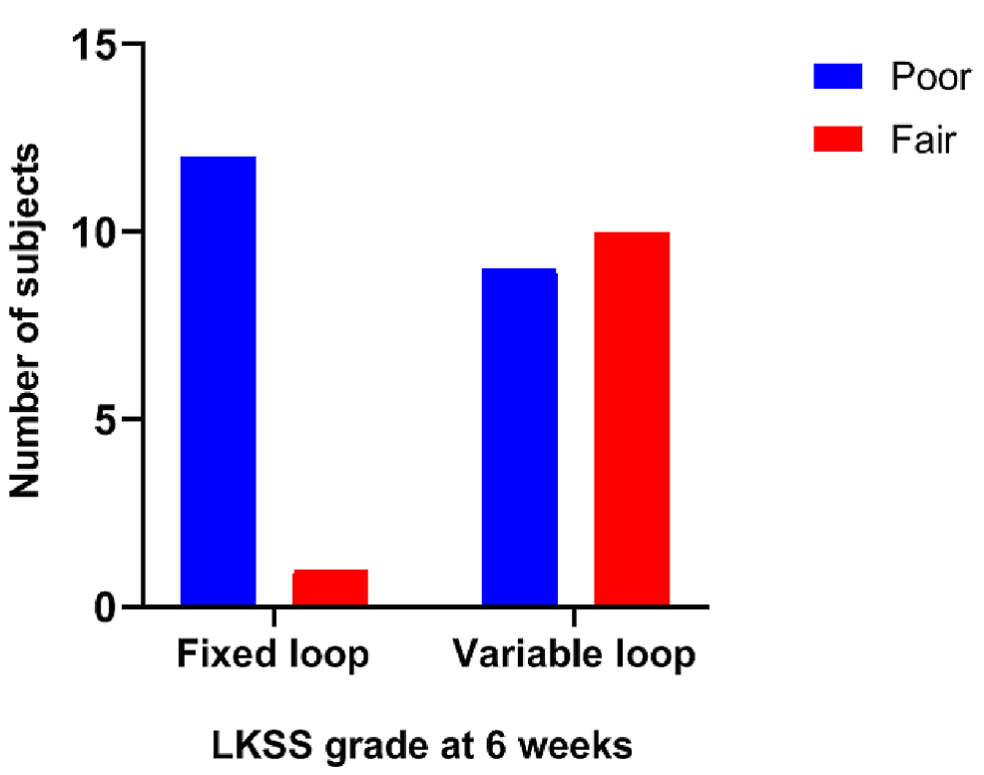
Lysholm knee scoring scale grade at 6 weeks LKSS, Lysholm knee scoring scale.

The Lysholm knee scoring scale was found to be comparable in both the groups, except at six weeks when it was graded as fair in 10 cases in the adjustable-loop group and only one patient in the fixed-loop group (*p*=0.009) (Figures 2-3).

One patient in each group had graft laxity at the end of six months and one year, respectively, as assessed by comparing the results of Lachman and anterior drawer tests on the affected and contralateral normal knees (Table 2).

**Table 2:** Tests for graft laxity *Negative or positive graft laxity refers to the absence or presence of laxity, respectively, relative to the normal contralateral knee.

Tests at 6 months	Fixed-loop group, *n* (%)	Variable-loop group, *n* (%)
Negative*	12 (92.3%)	18 (94.7%)
Positive*	1 (7.7%)	1 (5.3%)
Tests at 1 year		
Negative	12 (92.3%)	12 (92.3%)
Positive	1 (7.7%)	1 (7.7%)

**Figure 4:**
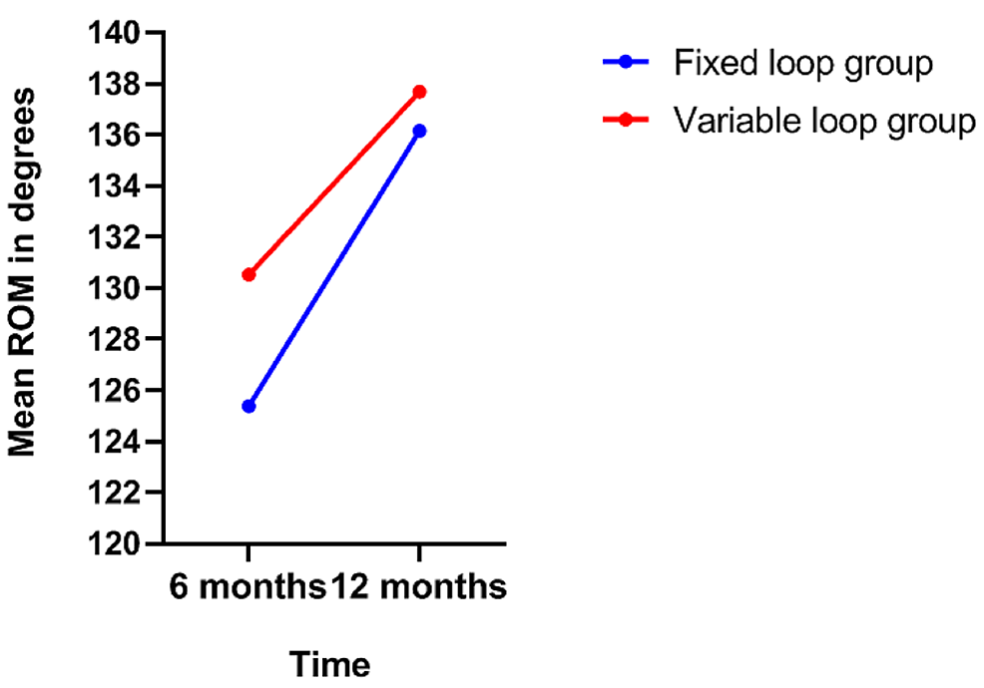
Range of motion ROM, range of motion.

The mean ROM at six months was 125.38$\pm$18.08 in the fixed-loop group and 130.53$\pm$17.15 in the variable group. The mean ROMs for the fixed- and variable-loop groups at the end of one year were 136.15$\pm$8.70 and 137.69$\pm$8.32, respectively (Figure 4).

**Figure 5:**
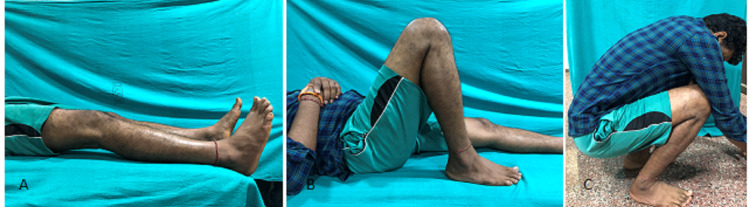
Patient on whom variable loop was used (at 6 months of follow-up): (A) extensor lag of 20 degrees; (B) knee flexion up to 120 degrees; (C) squatting slightly impaired

**Figure 6:**
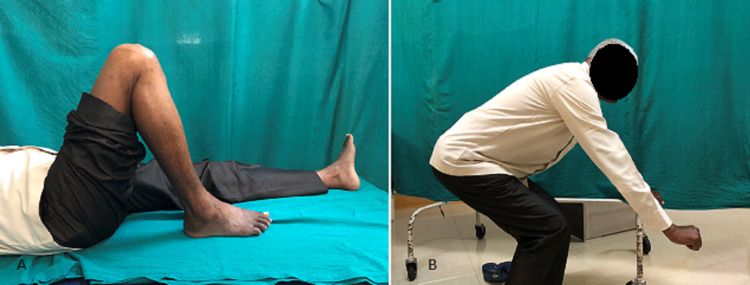
Patient on whom fixed loop was used (at 1 year of follow-up): (A) knee flexion up to 110 degrees; (B) inability to squat beyond 90 degrees

Knee stiffness, as evidenced by persistent extensor lag in one patient in the adjustable-loop group and incomplete flexion in one patient each in both the groups, was the only complication reported in our study, apart from graft laxity (Figures 5-6).

## Discussion

In this study, we compared the functional outcomes of arthroscopic ACL reconstruction with closed- and adjustable-loop devices in terms of clinically assessed laxity of the graft and Lysholm knee scores. Although several biomechanical studies have been conducted comparing the two fixation methods, we came across very few comparative clinical studies. In such a scenario, our study holds relevance.

The mean ages of patients in the fixed- and variable-loop groups were 26.1$\pm$11.0 and 25.8$\pm$11.7 years, respec­tively, in a study conducted by Boyle et al. [6]. The mean ages of patients in our study were 34.5$\pm$11 and 34.1$\pm$9.1 years in the fixed- and variable-loop groups, respectively.

Almost 61.5% and 68.4% of cases in the fixed- and variable-loop groups, respectively, had an associated meniscal injury. Weight-bearing was delayed by four weeks in all the patients who underwent meniscal repair (two patients in the fixed-loop group and six in the variable-loop group). Hagino et al. diagnosed an associated meniscal tear in 437 out of 552 knees with an ACL injury, on arthroscopic examination. The overall incidence of associated meniscal tear was thus found to be 79.2% [7].

Lanzetti et al. noted Lysholm knee scores of 92.8 and 93.2 in the fixed- and variable-loop groups, respectively, at the end of 12 months [8]. Wise et al. found that the mean Lysholm knee scores after surgery were 91.31 in the fixed-loop group and 87.28 in the vari­able-loop group [1]. Our study revealed similar results in terms of the Lysholm knee score. The mean scores at the end of one year were 91.54 and 91.69 in the fixed- and variable-loop groups, respectively, which meant that Lysholm knee scores were not considerably different in both groups at the end of the follow-up period although we observed a statistically significant difference at six weeks of follow-up.

Wise et al. compared functional outcomes of the fixed- and variable-loop groups based on the Tegner scale and 12-Item Short-Form Health Survey, apart from the Lysholm questionnaire [1]. Lanzetti et al. used the Lysholm knee score, International Knee Documentation Committee (IKDC) Subjective knee score and Tegner activity scale to compare the two groups [8]. Sharma and Parmar compared the functional outcomes of both groups based only on the Lysholm knee score, similar to our study [9]. To our knowledge, ours is the only study that compared the Lysholm knee scores of both groups periodically over six visits after surgery. Hence, we could assess the gradual improvement in the functional outcome of the operated cases over a period of one year. The fall in Lysholm knee scores immediately after surgery, when compared to the preoperative scores, is not of much significance as the patients gradually start performing their activities of daily living even as the graft heals. It takes about 8-12 weeks for soft tissue autografts to be incorporated into the femoral tunnel [10,11], with progressive graft healing occurring up to six months postoperatively [12]. We observed that the Lysholm knee score in the variable-loop group exceeded the preoperative score in just four weeks as opposed to three months in the fixed-loop group. The relevance of this finding needs to be dwelled upon.

The Lysholm knee score is one of the oldest knee-specific scoring systems used to assess patient-reported outcomes after ACL reconstruction [13]. A study conducted by Briggs et al. on over 1000 ACL reconstructed patients showed that the Lysholm scoring system had acceptable test-retest reliability and internal consistency [14]. The concise, yet informative nature of the Lysholm scoring scale has made it one of the most commonly used knee-specific scores [15]. Hence, we were justified in using it as the sole measure to assess patient-reported outcome scores.

Wise et al. assessed graft laxity using a KT-1000 arthrometer. Clinical laxity of knees, as defined by KT-1000 >3 mm [16-18], was observed in 12.5% and 6.1% of patients in the fixed- and variable-loop groups, respectively. The graft re-rupture rate observed was 8.7% in the fixed-loop group and 4.7% in the variable-loop group [1]. Sharma and Parmar found that one case (5%) in the fixed-loop group and three cases (15%) in the adjustable-loop group showed clinical laxity by the Lachman test, and 5% of cases in the closed-loop group and 10% in the adjustable-loop group had Grade 2+ rotatory laxity by the pivot shift test at the end of six months [9]. However, the results of both studies were not statistically significant. In our study, we assessed graft laxity clinically by comparing the findings of Lachman and anterior drawer tests of the affected knee with those of the normal knee, as the use of an arthrometer was not feasible. Both the groups had one patient each with graft laxity. Thus, our study also revealed a statistically insignificant difference between the two groups but had a longer follow-up period of one year. Moreover, we did not perform the pivot shift test during follow-up visits as it is best tested under spinal anaesthesia and gives ambiguous results otherwise [19,20].

Apart from graft laxity, the only other complication encountered was knee stiffness. Persistent extensor lag was noticed at six months in one patient in the variable-loop group and incomplete flexion was observed in one patient each in both the groups. Knee stiffness could be because of improper tunnel placement or non-compliance with the rehabilitation protocol [21,22]. A tibial tunnel that is too anteriorly placed can cause extensor lag [23,24]. Aggressive physiotherapy has to be initiated at the earliest for extensor lag as it could eventually result in quadriceps weakness, patellofemoral pain and gait abnormalities [21,25].

Lanzetti et al. found no difference in femoral tunnel enlargement on CT evaluation of knees in which ACL reconstruction was performed using fixed- and variable-loop constructs, 12 months after the surgery [8]. Hoher et al. recommended bone tunnel size measurement routinely post-ACL reconstruction as tunnel enlargement occurs due to longitudinal (bungee effect) and transverse (windshield wiper effect) motion of the graft within the tunnel [26]. However, we were unable to compare the two groups radiologically after the surgery, and this is one of the major limitations of our study.

Graft healing is the most important factor influencing the outcome of ACL reconstruction. The factors influencing graft healing include the graft type, tunnel length and orientation, length of graft inside the tunnel, disparity between the diameters of the tunnel and the graft, graft tension, graft motion in the tunnel and type of fixation [3]. We have only compared two modes of fixation in our study, without emphasizing the role of other contributory factors.

Several biomechanical studies have purported that variable-loop designs lengthen or slip, thereby delaying graft incorporation and causing knee instability [27,28]. Petre et al. postulated that graft re-tensioning following tibial fixation compensated for its slippage in variable-loop designs [27]. However, Johnson et al. noticed graft slippage despite secondary tensioning [4]. We performed graft re-tensioning after tibial fixation in all patients of the variable-loop group and observed no difference in graft laxity between the groups. This could be interpreted in several ways: (1) Re-tensioning reduces the chances of graft slippage. (2) As variable-loop designs allow more grafts to be pulled into the tunnel, graft slippage may be compensated for by the increased graft initially present in the tunnel. (3) Increased slippage observed in biomechanical studies could be because the force exerted is much more than that which the knee experiences after ACL reconstruction. This raises doubts as to whether the results obtained from biomechanical studies could be applied to the clinical setting.

Our results were consistent with the findings of Boyle et al., who conducted a retrospective comparative study to ascertain whether adjustable-loop devices loosen after ACL reconstruction. They concluded that there was no statistically significant variation in the graft failure rate between fixed- and variable-loop designs up to two years post-ACL reconstruction [6].

The fact that clinical studies like ours have not been demonstrating increased graft slippage in variable-loop designs as opposed to fixed-loop designs, as claimed by numerous biomechanical studies, could lead us to the fair assumption that biomechanical studies do not accurately represent clinical scenarios.

Our study has a few limitations. Assessment of graft laxity might be biased due to the subjective nature of clinical assessment. Moreover, postoperative CT and MRI evaluations could not be done to assess the comparative femoral tunnel widening and the relative amount of graft in the femoral tunnel, respectively. Furthermore, the problem of relatively small sample size was compounded by the fact that six patients in the variable-loop group were lost to follow-up after six months.

## Conclusions

Our study showed no statistically significant difference in laxity of the graft or functional outcomes of arthroscopic ACL reconstruction with fixed- and variable-loop devices, except for a better patient-reported outcome score in the variable-loop group at six weeks of follow-up. Although many biomechanical studies have shown variable-loop designs to exhibit more displacement than fixed-loop designs, there was no significant difference in the displacement of the device between the two groups in our study. Hence, there is a need for more comparative clinical studies in this direction.

## References

[1] Wise BT, Patel NN, Wier G, Labib SA (2017). Outcomes of ACL reconstruction with fixed versus variable loop button fixation. Orthopedics.

[2] Chang CB, Choi JY, Koh IJ, Lee KJ, Lee KH, Kim TK (2011). Comparisons of femoral tunnel position and length in anterior cruciate ligament reconstruction: modified transtibial versus anteromedial portal techniques. Arthroscopy.

[3] Ekdahl M, Wang JH, Ronga M, Fu FH (2008). Graft healing in anterior cruciate ligament reconstruction. Knee Surg Sports Traumatol Arthrosc.

[4] Johnson JS, Smith SD, LaPrade CM, Turnbull TL, LaPrade RF, Wijdicks CA (2015). A biomechanical comparison of femoral cortical suspension devices for soft tissue anterior cruciate ligament reconstruction under high loads. Am J Sports Med.

[5] Hong SH, Choi JY, Lee GK, Choi JA, Chung HW, Kang HS (2003). Grading of anterior cruciate ligament injury. Diagnostic efficacy of oblique coronal magnetic resonance imaging of the knee. J Comput Assist Tomogr.

[6] Boyle MJ, Vovos TJ, Walker CG, Stabile KJ, Roth JM, Garrett WE Jr (2015). Does adjustable-loop femoral cortical suspension loosen after anterior cruciate ligament reconstruction? A retrospective comparative study. Knee.

[7] Hagino T, Ochiai S, Senga S (2015). Meniscal tears associated with anterior cruciate ligament injury. Arch Orthop Trauma Surg.

[8] Lanzetti RM, Monaco E, De Carli A (2016). Can an adjustable-loop length suspensory fixation device reduce femoral tunnel enlargement in anterior cruciate ligament reconstruction? A prospective computer tomography study. Knee.

[9] Sharma B, Parmar RS (2018). Early outcome analysis of arthroscopic anterior cruciate ligament reconstruction using fixed closed loop and adjustable loop techniques: a prospective case series. J Orthop Allied Sci.

[10] Conner CS, Perez BA, Morris RP, Buckner JW, Buford WL Jr, Ivey FM (2010). Three femoral fixation devices for anterior cruciate ligament reconstruction: comparison of fixation on the lateral cortex versus the anterior cortex. Arthroscopy.

[11] Kawakami H, Shino K, Hamada M (2004). Graft healing in a bone tunnel: bone-attached graft with screw fixation versus bone-free graft with extra-articular suture fixation. Knee Surg Sports Traumatol Arthrosc.

[12] Rodeo SA, Arnoczky SP, Torzilli PA, Hidaka C, Warren RF (1993). Tendon-healing in a bone tunnel. A biomechanical and histological study in the dog. J Bone Joint Surg Am.

[13] Tegner Y, Lysholm J (1985). Rating systems in the evaluation of knee ligament injuries. Clin Orthop Relat Res.

[14] Briggs KK, Lysholm J, Tegner Y, Rodkey WG, Kocher MS, Steadman JR (2009). The reliability, validity, and responsiveness of the Lysholm score and Tegner activity scale for anterior cruciate ligament injuries of the knee: 25 years later. Am J Sports Med.

[15] Makhni EC, Padaki AS, Petridis PD (2015). High variability in outcome reporting patterns in high-impact ACL literature. J Bone Joint Surg Am.

[16] Daniel DM, Stone ML, Dobson BE, Fithian DC, Rossman DJ, Kaufman KR (1994). Fate of the ACL-injured patient. A prospective outcome study. Am J Sports Med.

[17] Tyler TF, McHugh MP, Gleim GW, Nicholas SJ (1999). Association of KT-1000 measurements with clinical tests of knee stability 1 year following anterior cruciate ligament reconstruction. J Orthop Sports Phys Ther.

[18] Daniel DM, Stone ML, Sachs R, Malcom L (1985). Instrumented measurement of anterior knee laxity in patients with acute anterior cruciate ligament disruption. Am J Sports Med.

[19] Lopomo N, Signorelli C, Rahnemai-Azar AA (2017). Analysis of the influence of anaesthesia on the clinical and quantitative assessment of the pivot shift: a multicenter international study. Knee Surg Sports Traumatol Arthrosc.

[20] Nakamura K, Koga H, Sekiya I (2017). Evaluation of pivot shift phenomenon while awake and under anaesthesia by different manoeuvres using triaxial accelerometer. Knee Surg Sports Traumatol Arthrosc.

[21] Tjoumakaris FP, Herz-Brown AL, Bowers AL, Sennett BJ, Bernstein J (2012). Complications in brief: anterior cruciate ligament reconstruction. Clin Orthop Relat Res.

[22] Petsche TS, Hutchinson MR (1999). Loss of extension after reconstruction of the anterior cruciate ligament. J Am Acad Orthop Surg.

[23] Allum R (2003). Complications of arthroscopic reconstruction of the anterior cruciate ligament. J Bone Joint Surg Br.

[24] Goble EM, Downey DJ, Wilcox TR (1995). Positioning of the tibial tunnel for anterior cruciate ligament reconstruction. Arthroscopy.

[25] Eckenrode BJ, Carey JL, Sennett BJ, Zgonis MH (2017). Prevention and management of post-operative complications following ACL reconstruction. Curr Rev Musculoskelet Med.

[26] Höher J, Möller HD, Fu FH (1998). Bone tunnel enlargement after anterior cruciate ligament reconstruction: fact or fiction?. Knee Surg Sports Traumatol Arthrosc.

[27] Petre BM, Smith SD, Jansson KS (2013). Femoral cortical suspension devices for soft tissue anterior cruciate ligament reconstruction: a comparative biomechanical study. Am J Sports Med.

[28] Barrow AE, Pilia M, Guda T, Kadrmas WR, Burns TC (2014). Femoral suspension devices for anterior cruciate ligament reconstruction: do adjustable loops lengthen?. Am J Sports Med.

